# 

**Published:** 1915-01-30

**Authors:** 


### Welsh Hospital, Netley.

A deputation, consisting of the Earl of Plymouth, Alderman James Robinson (ex-Lord Mayor of Cardiff), Sir William James Thomas (honorary treasurer), Mr. J. A. Jones, Colonel A. W. Sheen, and Mr. Cyrus J. Evans, recently visited the War Office in regard to the Welsh Hospital, and was received by Sir Alfred Keogh, Director-General of the Army Medical Service. The question of extending the work of the hospital was discussed, and ultimately it was decided that the best service which could be rendered immediately would be for the hospital to increase the accommodation at Netley by another 100 beds. It has been arranged that the hospital shall be taken abroad at a very early date, and the committee is proceeding meanwhile to make the extension, which will double the accommodation.

### A GLASGOW MEDICAL DRAMATIST.

Dr. John Wain Findlay, of Glasgow, son of Dr. William Findlay, of the same city, and brother of Dr. Findlay, of the Children's Hospital, died recently at his home, Lyttle Park, Lanarkshire, at the early age of forty-one, deeply regretted alike as a physician and a dramatist. He came of a gifted family, and had a long list of works to his credit. His father was known in the literary world as "George Umber," and other members of the family have distinguished themselves in various branches of art. One of his brothers is a painter.

### Workers' Acknowledgment to Sir Edward Wood.

On the occasion of his seventy-sixth birthday on January 16, Sir Edward Wood was the recipient of an interesting presentation from the working people of the town and county of Leicester, when an illuminated address was handed to him expressing the gratitude of the workers for his great work in the reconstruction, remodelling, and extension of the Leicester Royal Infirmary, and in the establishment of convalescent homes in the county for men and women workers.

The address was accompanied by copies of two famous pictures, "The Good Samaritan," from the Leicester Art Gallery, and Luke Fildes' "The Doctor," duplicates of which will subsequently be presented to the Leicester Royal Infirmary.

This spontaneous expression on the part of the workpeople must undoubtedly be very gratifying to Sir Edward Wood, and all interested in hospital work will join in the hope that his health may soon enable him to resume attendance and assist in the deliberations of the committees of the charitable institutions in Leicestershire with which his name is associated.

### Personalia.

Dr. G. N. Bartlett, second assistant medical officer at Horton Mental Hospital, has resigned his position on his appointment to be medical superintendent of the Exeter City Mental Hospital.

Professor James Swain, M.D., who is senior surgeon at the Bristol Royal Infirmary, has been appointed by the War Office hon. consulting surgeon to the troops of the Southern Command.

Mr. Harold V. Pinkham, clerk at the Cancer (Free) Hospital, Fulham Road, has joined the 9th (2nd Reserve Battalion) County of London Regiment, and therefore will not resume his duties at the hospital until the end of the war.

### SAVILL PRIZE, 1914.

The examiners have recommended Mr. George Riddoch, M.B., Ch.B., for this prize. Mr. Riddoch was first man in his year at Aberdeen University, where he graduated in 1913 with first-class honours, gaining the Murray Medal and Scholarship. He also gained the Fife Jamieson Medal, which is awarded to the most distinguished student in the anatomy department, and acted as junior demonstrator in the University. The subject of the thesis set in connection with this prize was Intracranial and Intraspinal Neoplasms.

### "THE WEST HAM AND SOUTH ESSEX HOSPITAL."

Mr. Joseph Gould, of Forest Lane, Stratford, E., who left estate of the gross value of £23,076, has left all his securities of the West Ham Corporation to his sister, Amelia Jane Gould, for life, with remainder to the West Ham and South Essex Hospital (*sic*).

### Glamorgan and Monmouth Hospital in France.

In less than seven weeks from the first committee meeting the Glamorgan and Monmouthshire Hospital for the French Army is in full working order, and patients have been received. The Hotel de Russie at Berck Plage was taken over on December 12 and thoroughly cleaned. Two rooms were selected for the operating theatre and dressing room, and have been transformed into equipped rooms wherein any major or minor operation may be performed. An adjacent room is used for radiography. The difficulty of distributing the beds has been overcome, as also the kindred problems of organising the kitchen arrangements to meet the requirements of some 140 persons, providing messrooms for the staff, and of dealing with soiled and muddy clothing. Dr. H. G. Cook, Mrs. Marcus Paterson, and their staff have been responsible for the arrangements. Funds amounting to over £4,000 have already been collected, but further subscriptions are needed.

### ORDER OF ST. JOHN OF JERUSALEM.

The King has sanctioned the following, among other, promotions in and appointments to the Order of the Hospital of St. John of Jerusalem in England:—As Knight of Grace: Lieut.-Colonel William Ernest Jennings, M.D., D.P.H., I.M.S. (from Honorary Serving Brother).

RATES OF SUBSCRIPTION : Twelve months, 6/6 ; Six months, 4/- ; Three months, 2/-, post free to any address in the United Kingdom. Abroad, Twelve months, 8/8 ; Six months, 5/- ; Three months, 2/6, post free.—Address The Subscription Dept., "THE HOSPITAL," The Hospital Building, 28/29 Southampton Street, Strand, London, W.C.

